# Ghana’s National Health Insurance enrollment: Does the intersection of educational and residential status matter?

**DOI:** 10.1371/journal.pone.0318202

**Published:** 2025-02-21

**Authors:** Roger Antabe, Florence W. Anfaara, Yujiro Sano, Daniel Amoak

**Affiliations:** 1 Department of Health and Society, University of Toronto Scarborough, Toronto, Ontario, Canada; 2 Department of Gender, Sexuality, and Women’s Studies, Western University, London, Ontario, Canada; 3 Department of Sociology and Anthropology, Nipissing University, North Bay, Ontario, Canada; 4 Department of Geography and Environmental Management, University of Waterloo, Waterloo, Ontario, Canada; Aminu Kano Teaching Hospital, NIGERIA

## Abstract

**Background:**

Since its inception in 2003, Ghana’s Health Insurance Scheme (NHIS) has received considerable scholarly attention on the determinants of enrollment. While most of these studies highlight the role of some socioeconomic and geographical factors, no study has explored the intersection of educational attainment and residence on NHIS enrollment. We aim to contribute to the literature and health policy in Ghana by examining the intersection of educational attainment and rural-urban residence on NHIS enrollment among women and men.

**Methods:**

We used nationally representative data from the 2022 Ghana Demographic and Health Survey (GDHS). Using STATA 17, we applied multivariable logistic regression to our analytical sample comprising women (n = 14997) and men (n = 7040).

**Results:**

Overall, we found that more women (90%) than men (73%) enrolled on the NHIS. Adjusting for a range of control variables, we found that women and men with secondary (OR: 1.61, 95% CI: 1.28–2.02; OR: 1.45, 95% CI: 1.16–1.82) and higher education (OR: 1.81, 95% CI: 1.24–2.64; OR: 2.85, 95% CI: 2.03–3.99) were more likely to have enrolled into the NHIS compared to those with no formal education. This difference was particularly heightened among women and men with no education. Rural women (96%) and men (90%) with higher education had higher enrollment rates compared to their urban counterparts.

**Conclusion:**

We recommend revising the NHIS equity and pro-poor policy to include vulnerability at the intersection of low educational attainment and rural residence.

## Introduction

In 2003, Ghana established the National Health Insurance Scheme (NHIS) to promote equity by making healthcare services accessible to the poor, vulnerable, and structurally disadvantaged populations [1,2]. As a policy objective, the NHIS is expected to lessen or remove financial and economic barriers to healthcare access and utilization among Ghanaians. This has resulted in continuous policy changes that target the enrollment of society’s poorest and most vulnerable [3,4]. Given the NHIS’ equity and pro-poor policy initiatives, an overwhelming majority of existing studies on the NHIS have examined the impact of people’s socioeconomic status, particularly household wealth and income, on enrollment [5,6]. While the findings from these studies are revealing, nascent research has yet to explore how other critical socioeconomic factors, such as educational attainment, may further interact with people’s geographical characteristics to influence NHIS registration. The objective of our study is to examine how educational attainment and geographical characteristics (i.e., place of residence) intersect to affect NHIS enrollment in Ghana. We expect our findings to inform the revision of the NHIS equity and pro-poor enrollment policy to include other forms of vulnerability at the intersection of educational attainment and geographical attributes.

As a core mandate to achieving its health equity goal, the NHIS targets financially poor and marginalized Ghanaians including women and indigents through its premium payment scheme. The premium is commensurate with people’s economic conditions. The poor are made to pay lower premiums than their well-to-do counterparts, who are required to pay more [7]. For example, to improve maternal health outcomes for vulnerable women who cannot afford quality maternal and postnatal services, the NHIS offers a free maternal healthcare policy (FMHP) to remove the financial barriers to maternal and child healthcare services [8,9]. In addition, indigents are exempted from NHIS premium payment and renewal. The NHIS defines indigents as people who a) have no recognized source of income, b) are unemployed, and c) have no recognized place of abode [2,4]. Beneficiaries of the Livelihood Empowerment Against Poverty (LEAP), are registered in the NHIS as indigents. LEAP is a government of Ghana’s flagship program aimed at reducing the social impacts of extreme poverty [10]. Children below the age of 18, those aged 70 + years, pensioners, and people with severe mental health conditions also receive free registration and renewal under the scheme [ 2,11,12].

It is noteworthy that several empirical studies have documented the immense contribution of the NHIS’ equity and pro-poor policy in increasing access and utilization of health services in Ghana. One of such is Mensah et al. [13] who note that under the FMHP, pregnant women were more likely to deliver at a hospital, have a professionally assisted delivery and experience fewer birth complications. Twum et al. [8] also observed that pregnant women enrolled in the NHIS were 39.5 times more likely to meet the six recommended maximum antenatal care visits, and 2.6 times more likely to meet an average of four. Further, to assess the relationship between NHIS and access to health services, Ameyaw et al. [14] found pregnant women’s perception of NHIS enrolment to be positive. For instance, over 78% believed that the NHIS was helping pregnant women access healthcare services. The NHIS pro-poor policy does not only target women and indigents, but also youth and migrant teenagers who may be experiencing homelessness. For example, in the 2018 National Health Insurance Authority (NHIA) report, about 47% of active members were youth under 18 years [15]. For migrant teenage head porters, Alatinga et al. [12] found that those who enjoyed premium exemptions were more likely to maintain active NHIS membership.

While removing financial barriers to NHIS enrolment through registration fees and renewal exemptions is critical to meeting the healthcare needs of many Ghanaians, we argue that financial barriers alone are insufficient to address the health equity gaps inherent in healthcare access and utilization. Indeed, some scholars have critiqued the current approach to identifying the poorest and most vulnerable for NHIS enrollment as inadequate. Jehu-Appiah et al. [16] argue for a more efficient and equitable approach that will cover the hitherto excluded poor people that the current premium exemption neglected. Among other things, the authors suggested a three-pronged approach, which include Proxy Means Testing (PMT) for relatively low poverty-incidence urban settings, Participatory Welfare Ranking (PWR) in relatively low poverty-incidence rural settings and Geographic Targeting (GT) in high poverty-incidence settings [16]. While PMT expands the poverty classification to include indicators of household welfare factors, ownership of selected assets, and other social characteristics, GT and PWR target assessing poor people on the basis of group dynamics and locality as well as the communities’ own definition of their status [16, p. 169]. According to Kumi-Kyereme and Amo-Adjei [17], PMT and GT are useful approaches for identifying populations requiring particular interventions to increase NHIS enrollment. Furthermore, Antabe and colleagues [2] established in the Upper West Region of Ghana that those who reported any form of household food insecurity were less likely to enroll. The authors called for a reassessment of the approaches to identifying the poor and vulnerable, as the current strategies may be exclusionary.

Based on this, it is possible that the NHIS may not meet its equity and pro-poor mandates unless other barriers are identified and addressed [1,12]. However, despite these scholars’ growing concerns, studies have only prioritized understanding the impact of household wealth and income as key socioeconomic indicators of NHIS enrollment. Little attention has been paid to other crucial indicators of socioeconomic status, such as educational attainment, and how it may intersect with the geographical characteristics of Ghanaians to impact enrollment. Therefore, our study’s specific objective is to examine how the intersection of educational attainment with place of residence is associated with NHIS enrollment. Our findings will contribute to the discussion on extending the NHIS equity and pro-poor policy mandate beyond financial vulnerability to include enrollment barriers at the intersection of educational attainment and geographical characteristics.

## Methods

The Ghana Demographic and Health Survey (GDHS) is reliable in providing various demographic and health metrics estimates, including insights into Ghana’s NHIS. Data was collected from 17 October 2022 to 14 January 2023. The survey employed a multi-stage cluster sampling method. In the initial stage, clusters were selected based on their size, and in the subsequent stage, a set number of households (approximately 25–30) were systematically randomly chosen from each cluster. Within the surveyed households, 15,317 women aged 15–49 were deemed eligible for individual interviews. The GDHS successfully completed interviews with 15,014 of these women, resulting in a response rate of 98%. For the male survey, 7,263 men aged 15–59 were identified as eligible, with 7,044 completing the interview, yielding a 97% response rate. Given that missing data represented less than one percent, our analysis applied a listwise deletion method. This process resulted in final analytical samples of 14,997 women and 7,040 men. The GDHS uses the Ghana Statistical Service’s definition of locality, which states that an urban locality has 5,000 or more residents, while any place with less than 5,000 inhabitants is rural [18]. Additionally, the GDHS has created its own household wealth index to guide researchers in measuring household wealth. The survey protocol, such as the biological measurement and test procedures, was appraised and approved by the Ghana Statistical Service and the Institutional Review Board of ICF, Rockville, Maryland, USA. Interviewers obtained informed consent by reading the consent statement of the respondents, who may accept or decline to participate.

### Measures

The dependent variable measures whether respondents are enrolled in the NHIS (0 = no; 1 = yes). We have one primary independent variable, including level of education (0 = no education; 1 = primary education; 2 = secondary education; 3 = higher education). We further introduce a series of control variables, such as place of residence (0 = urban; 1 = rural), household wealth (0 = poorest; 1 = poorer; 2 = middle; 3 = richer; 4 = richest), employment (0 = unemployed; 1 = employed), age (0 = 15-19; 1 = 20-24; 2 = 25-29; 3 = 30-34; 4 = 35-39; 5 = 40-44; 6 = 45-49; 7 = 50-54; 8 = 55-59), marital status (0 = never married; 1 = currently married; 2 = formerly married), and religion (0 = Chrisitan; 1 = Muslim; 2 = traditionalist; 3 = no religion).

### Statistical analysis

We initially employed descriptive analysis to understand the characteristics of women and men. For logistic regression analysis, we built four models each for women and men. First, the full sample model assessed the overall relationship between NHIS enrollment and various predictors, enabling us to explore whether education is associated with NHIS enrollment among women and men. The urban model focused specifically on urban areas, serving as a platform for us to examine whether the relationship between education and NHIS enrollment is significant among women and men in urban areas. In contrast, the rural model examined the same predictors within rural areas. Finally, the interactive model explored the interaction between education and place of residence, offering insights into whether the association between education and NHIS enrollment varies between urban and rural contexts. All regression analysis controlled for a range of control variables, such as household wealth, employment, age, marital status, and religion. All analysis used STATA 17 (State Corp, College Station, TX, USA). The ‘svy’ function was applied in statistical analysis to adjust for the cluster sampling design and sampling weights.

## Results

Table 1 presents the characteristics of the study sample, which includes 7,040 men and 14,997 women. Among men, 73% were enrolled in the NHIS compared to 90% of women. Regarding education, 11% of men and 16% of women had no formal education, while 14% of men and 10% of women had higher education. Urban residents constituted 55% for men and 57% for women. Employment rates show that 84% of men were employed versus 75% of women. In terms of marital status, 49% of men were currently married at the time of data collection compared to 55% of women, while 10% of women were formerly married, a higher proportion than men (i.e., 5%). Regarding religion, 77% of women and 70% of men identify as Christian, while Muslims make up 19% of women and 21% of men. Traditionalists and those with no religion are a minority in both groups.

**Table 1:** Sample characteristics.

	Percentage	
	Men (n = 7,040)	Women (n = 14,997)
**NHIS enrollment**		
No	27	10
Yes	73	90
**Education**		
No education	11	16
Primary education	12	14
Secondary education	63	60
Higher education	14	10
**Place of residence**		
Urban	55	57
Rural	45	43
**Household wealth**		
Poorest	18	16
Poorer	18	18
Middle	18	21
Richer	23	23
Richest	23	23
**Employment**		
Unemployed	16	25
Employed	84	75
**Age**		
15–19	20	18
20–24	15	18
25–29	13	16
30–34	12	15
35–39	11	14
40–44	10	11
45–49	8	9
50–54	6	NA
55–59	5	NA
**Marital status**		
Never married	46	35
Currently married	49	55
Formerly married	5	10
**Religion**		
Christian	70	77
Muslim	21	19
Traditionalist	4	2
No religion	5	2
Total	100	100

**Fig 1:**
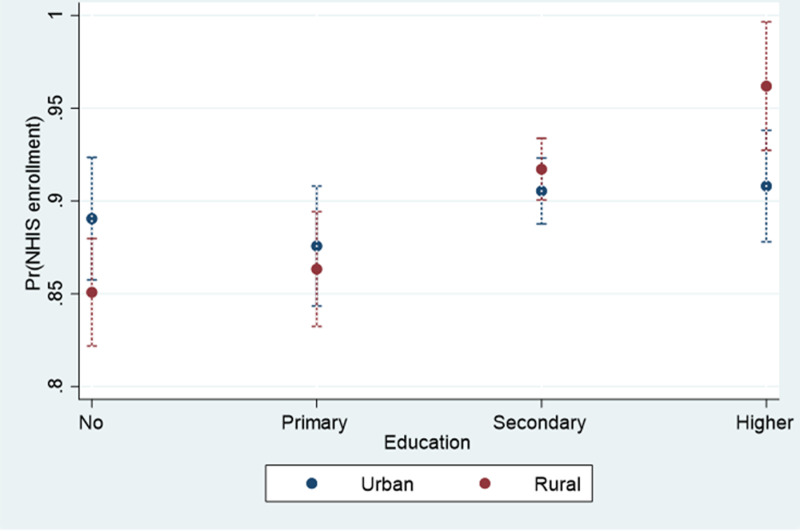
Predictive margins of education and place of residence among women.

Table 2 shows findings from logistic regression among women. In the full sample, higher educational attainment was strongly associated with increased NHIS enrollment, even after adjusting for a range of control variables. Women with secondary education had an odds ratio (OR) of 1.61 (95% CI: 1.28–2.02), while those with higher education had an even higher OR of 1.81 (95% CI: 1.24–2.64). This trend persisted across both urban and rural settings, though the impact of education was more pronounced in rural areas. Specifically, in the rural model, women with higher education had a markedly higher likelihood of NHIS enrollment (OR: 4.15, 95% CI: 1.76–9.75) compared to their urban counterparts, where the effect was less pronounced (OR: 1.28, 95% CI: 0.78–2.11). The interaction model further highlighted that the benefits of secondary and higher education on NHIS enrollment were significantly enhanced in rural areas, with an OR of 1.67 (95% CI: 1.09–2.56) and 3.72 (95% CI: 1.24–11.17), respectively. In other words, as illustrated in Fig 1, the difference in NHIS enrollment between urban and rural areas was most pronounced among women with no formal education, with enrollment rates of 89% in urban areas compared to 85% in rural areas. However, this trend had reversed for women with secondary and higher education: those with secondary education had enrollment rates of 91% in urban areas versus 92% in rural areas, and those with higher education had enrollment rates of 91% in urban areas compared to 96% in rural areas.

**Table 2:** Regression analyses of NHIS enrollment among women in Ghana.

	Full sample model			Urban model			Rural model			Interactive model		
	OR	95% CI		OR	95% CI		OR	95% CI		OR	95% CI	
**Education**												
No education	1.00			1.00			1.00			1.00		
Primary education	1.05	0.84	1.30	0.86	0.57	1.28	1.16	0.89	1.51	0.86	0.59	1.28
Secondary education	1.61	1.28	2.02	1.18	0.78	1.77	2.02	1.52	2.68	1.18	0.80	1.74
Higher education	1.81	1.24	2.64	1.28	0.78	2.11	4.15	1.76	9.75	1.22	0.75	1.98
**Place of residence**												
Urban	1.00									1.00		
Rural	1.05	0.81	1.37							0.70	0.46	1.04
**Education** * **place of residence**												
Primary education*rural										1.28	0.82	2.01
Secondary education*rural										1.67	1.09	2.56
Higher education*rural										3.72	1.24	11.17
**Household wealth**												
Poorest	1.00			1.00			1.00			1.00		
Poorer	1.15	0.89	1.49	1.21	0.63	2.30	1.02	0.78	1.32	1.10	0.85	1.41
Middle	1.16	0.88	1.54	0.81	0.46	1.41	1.34	0.93	1.95	1.09	0.82	1.44
Richer	1.20	0.87	1.65	0.88	0.48	1.60	1.42	0.87	2.33	1.13	0.82	1.56
Richest	1.41	0.98	2.03	1.11	0.58	2.13	1.30	0.69	2.43	1.38	0.95	1.99
**Employment**												
Unemployed	1.00			1.00			1.00			1.00		
Employed	0.90	0.74	1.10	0.88	0.64	1.20	0.97	0.76	1.22	0.90	0.74	1.10
**Age**												
15–19	1.00			1.00			1.00			1.00		
20–24	1.25	0.99	1.58	1.38	0.98	1.95	1.13	0.83	1.55	1.26	1.00	1.59
25–29	1.11	0.82	1.51	0.90	0.58	1.38	1.60	1.07	2.39	1.12	0.83	1.52
30–34	1.13	0.80	1.61	0.92	0.54	1.54	1.53	1.00	2.33	1.14	0.81	1.61
35–39	1.06	0.75	1.49	0.99	0.59	1.66	1.18	0.77	1.79	1.07	0.76	1.50
40–44	0.99	0.69	1.43	0.95	0.55	1.62	1.07	0.67	1.72	0.99	0.69	1.43
45–49	0.70	0.49	0.99	0.58	0.35	0.96	0.89	0.56	1.42	0.70	0.50	1.00
50–54												
55–59												
**Marital status**												
Never married	1.00			1.00			1.00			1.00		
Currently married	2.39	1.91	3.00	2.60	1.90	3.56	2.11	1.56	2.86	2.39	1.91	2.99
Formerly married	1.49	1.11	1.99	1.69	1.12	2.55	1.20	0.81	1.76	1.47	1.10	1.97
**Religion**												
Christian	1.00			1.00			1.00			1.00		
Muslim	1.10	0.86	1.40	0.96	0.66	1.39	1.30	1.00	1.68	1.09	0.85	1.39
Traditionalist	0.44	0.29	0.65	0.41	0.16	1.06	0.50	0.32	0.77	0.46	0.31	0.69
No religion	0.59	0.36	0.99	0.55	0.24	1.27	0.62	0.33	1.18	0.60	0.36	1.01

OR, odds ratio; CI, confidence intervals.

**Fig 2:**
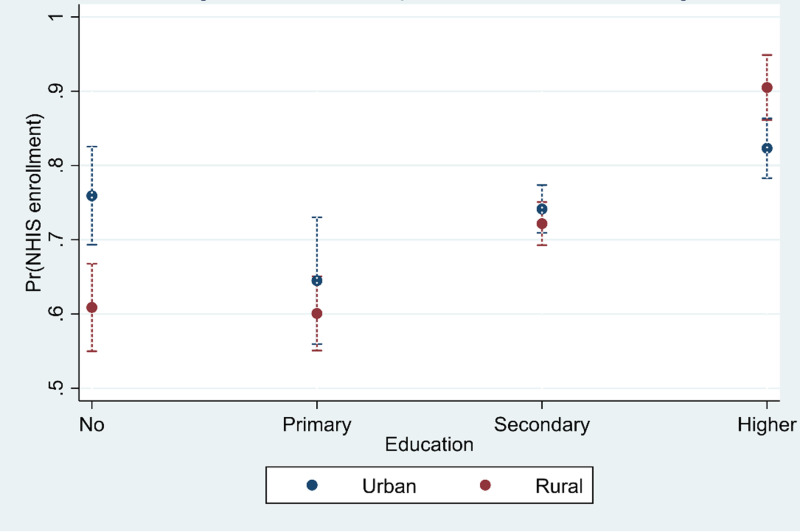
Predictive margins of education and place of residence among men.

Table 3 shows findings from logistic regression among men. Consistent with findings among women, higher education was strongly associated with an increased likelihood of NHIS enrollment. For the full sample, men with secondary education had an OR of 1.45 (95% CI: 1.16–1.82), while those with higher education had an even higher OR of 2.85 (95% CI: 2.03–3.99). This trend is particularly pronounced in rural areas. In the rural model, higher education significantly boosts the likelihood of NHIS enrollment with an OR of 7.36 (95% CI: 4.18–12.96), compared to an OR of 1.42 (95% CI: 0.84–2.39) in urban settings. The interactive model highlighted that the benefits of higher education on NHIS enrollment were notably greater in rural areas, with an OR of 4.47 (95% CI: 2.19–9.15), underscoring the critical role education plays in enhancing health insurance uptake where access might otherwise be limited. Put differently, largely consistent with women, as depicted in Fig 2, the disparity in NHIS enrollment between urban and rural areas was most significant among men with no formal education, with enrollment rates of 76% in urban areas versus 61% in rural areas. For those with secondary education, the enrollment rates were slightly higher in urban areas at 74%, compared to 72% in rural areas. Conversely, among men with higher education, enrollment rates were notably higher in rural areas at 90%, compared to 82% in urban areas.

**Table 3:** Regression analyses of NHIS enrollment among men in Ghana.

	Full sample model			Urban model			Rural model			Interactive model		
	OR	95% CI		OR	95% CI		OR	95% CI		OR	95% CI	
**Education**												
No education	1.00			1.00			1.00			1.00		
Primary education	0.85	0.65	1.10	0.55	0.33	0.91	1.01	0.76	1.34	0.56	0.34	0.92
Secondary education	1.45	1.16	1.82	0.87	0.56	1.36	1.83	1.42	2.36	0.90	0.61	1.35
Higher education	2.85	2.03	3.99	1.42	0.84	2.39	7.36	4.18	12.96	1.51	0.93	2.43
**Place of residence**												
Urban	1.00									1.00		
Rural	0.89	0.72	1.10							0.47	0.30	0.74
**Education** * **place of residence**												
Primary education*rural										1.74	0.97	3.10
Secondary education*rural										1.91	1.22	3.00
Higher education*rural										4.47	2.19	9.15
**Household wealth**												
Poorest	1.00			1.00			1.00			1.00		
Poorer	1.15	0.90	1.47	1.24	0.68	2.26	1.03	0.79	1.33	1.08	0.85	1.38
Middle	1.24	0.94	1.64	0.99	0.60	1.64	1.27	0.88	1.82	1.14	0.86	1.50
Richer	1.18	0.88	1.58	1.05	0.60	1.82	1.15	0.76	1.72	1.10	0.82	1.48
Richest	1.22	0.86	1.72	1.17	0.65	2.09	1.04	0.62	1.74	1.17	0.83	1.67
**Employment**												
Unemployed	1.00			1.00			1.00			1.00		
Employed	0.66	0.50	0.88	0.72	0.46	1.10	0.63	0.43	0.93	0.66	0.50	0.88
**Age**												
15–19	1.00			1.00			1.00			1.00		
20–24	0.67	0.51	0.89	0.78	0.49	1.23	0.59	0.41	0.85	0.68	0.51	0.89
25–29	0.35	0.25	0.49	0.33	0.19	0.59	0.36	0.26	0.51	0.35	0.25	0.49
30–34	0.33	0.24	0.46	0.34	0.20	0.57	0.32	0.21	0.48	0.33	0.24	0.46
35–39	0.30	0.21	0.42	0.26	0.15	0.44	0.34	0.21	0.53	0.30	0.21	0.42
40–44	0.27	0.19	0.40	0.23	0.13	0.42	0.32	0.20	0.51	0.28	0.19	0.40
45–49	0.49	0.33	0.74	0.47	0.24	0.90	0.51	0.31	0.87	0.50	0.33	0.75
50–54	0.51	0.35	0.76	0.41	0.21	0.79	0.64	0.40	1.04	0.52	0.35	0.77
55–59	0.49	0.31	0.75	0.51	0.24	1.05	0.47	0.27	0.80	0.49	0.32	0.76
**Marital status**												
Never married	1.00			1.00			1.00			1.00		
Currently married	1.20	0.97	1.48	1.20	0.90	1.59	1.21	0.90	1.62	1.19	0.97	1.47
Formerly married	1.04	0.76	1.43	1.07	0.64	1.78	1.04	0.70	1.54	1.02	0.74	1.40
**Religion**												
Christian	1.00			1.00			1.00			1.00		
Muslim	1.21	0.99	1.47	1.22	0.90	1.67	1.18	0.93	1.50	1.19	0.98	1.46
Traditionalist	0.73	0.53	1.01	0.35	0.20	0.62	0.98	0.68	1.42	0.75	0.54	1.04
No religion	0.33	0.24	0.45	0.27	0.17	0.42	0.38	0.25	0.57	0.33	0.24	0.45

OR, odds ratio; CI, confidence intervals.

## Discussion

In this study, we aimed to examine how the intersection of educational attainment and geographical characteristics affected the enrollment of women and men into Ghana’s NHIS program. Our findings show high levels of NHIS enrollment; with more women enrolling compared to men. Our findings are consistent with those of earlier studies in Ghana, which have not only established high levels of enrollment rates into the NHIS but the observation that more women tend to enroll relative to men [19,20]. While the gendered nature of NHIS enrollment may be partly explained by the free maternal health policy that exempts expectant mothers from paying for NHIS registrations, premiums and renewals, the high rates of enrollment at the general population level may suggest heightened trust in the scheme to create improved access to healthcare services in the country. The NHIS must continue to target more citizens, especially men, to enroll in the scheme.

The findings also indicate that educational attainment was associated with NHIS enrollment for women and men. Specifically, Ghanaians with higher levels of educational attainment were more likely to enroll on the scheme compared to their counterparts with no formal education. This finding is consistent with prior studies in Ghana, such as Salari et al. [19] and Kumi-Kyereme and Amo-Adjei [17], highlighting the vital role of educational attainment on NHIS enrollment. We note that secondary and higher educational attainment also positively impacted NHIS enrollment in rural and urban areas. However, the finding that urban residents are more likely to enroll than their rural colleagues may not be too surprising in the context of earlier research. These studies have discussed the barriers rural residents face in accessing basic social infrastructural services, including health services [21,22]. It is possible that the urban bias in the siting of health insurance offices and healthcare facilities may be working to discourage rural residents from enrolling, as they have to pay additional transportation costs to register at NHIS offices and to utilize health services with their membership [20,23,24].

We notice other dynamics of NHIS enrollment when place of residence interacts with educational attainment. Specifically, among women and men in rural areas, those with secondary and higher educational attainment were found to be more likely to have enrolled on the NHIS than their counterparts with no formal education. The lowest enrollment rates are reported for rural residents without formal education. Unpacking this finding, we point to two plausible ways. First, it can be argued that formal education may be more critical for NHIS enrollment in rural Ghana than for urban residents. While the rural penalty for registering for health insurance and accessing healthcare services is well established in the literature, those with formal educational attainment in rural areas may be better positioned to understand the benefits of NHIS enrollment [2,25]. Second, it is also possible that those with secondary and higher educational attainment in rural areas can easily digest information on the NHIS and appreciate the enrollment benefits. Our finding is consistent with Van Der Wielen et al. [26], who established that higher educational attainment was associated with enrollment in rural communities in Ghana. Similarly, among the rural poor in Ghana, it was found that increasing levels of education were associated with the uptake of health insurance enrollment [27]. This finding underscores the importance of understanding NHIS vulnerability beyond financial indicators to explore other socioeconomic and non-financial vulnerabilities that may be intersecting to influence enrollment.

The finding among men suggests that the unemployed lag behind their employed counterparts in NHIS enrollment. This may be pointing to the advantages of holding employment in Ghana. The NHIS deducts premium payments directly from the mandatory social insurance contributions of the employee, particularly those in formal workspaces [15]. With this dynamic, the employee may not feel the direct financial burden of paying out of pocket for insurance enrollment relative to their unemployed counterparts. Prior studies by Anarwat et al. [24] and Salari et al. [19] also found similar patterns between employment and enrollment, where unemployed people were less likely to register for health insurance. Among those at risk of statelessness in two districts in the Central Region of Ghana, Quartey et al. [28] explained that unemployment reduced the capacity of people to pay for premiums. The authors observed that this translated into a financial barrier to NHIS enrollment among the unemployed.

We further found that men in all other age categories were less likely to enroll in the NHIS than the youngest, aged 15–19. This finding is unsurprising in the context of the NHIS exemption policies, where children under 18 years are exempted from paying premiums [2,12,15]. This exemption policy may be working to increase enrollment for the youngest population relative to older age groups who are required to pay NHIS premiums. Finally, we note that women who reported being traditionalists and men who had no religion were less likely to have enrolled compared to their Christian counterparts. This finding is explained by Anfaara et al. [29], who contended that in Ghana, organized religion, particularly Christianity and Islam, frequently invites health experts to educate their congregants on health-related issues. Traditionalists and people with no religion may, therefore, miss valuable, informative sessions that shed light on critical health issues, such as the importance of NHIS enrolment.

Our study has some noteworthy limitations. First, the GDHS data was collected contemporaneously, meaning our findings are limited to statistical association and must be interpreted cautiously. Second, our selected control variables may not exhaustively describe all the factors influencing NHIS enrollment, including exposure to mass media and perceived quality [25,30]. Future studies can collect primary data that includes these variables to understand how they influence the relationship between the independent and dependent variables. Finally, the outcome variable did not ask if the enrolled were still active members of the NHIS. Follow-up studies can explore this relationship. Despite these limitations, our study is the first to examine how non-financial socioeconomic vulnerabilities, defined by educational attainment and rural-urban residence, influence NHIS enrollment.

## Conclusion

Our findings have some policy implications. The NHIS may have to revise and extend its equity and pro-poor mandate beyond financial vulnerability to include other barriers to enrollment, such as those with low educational attainment living in the country’s rural communities. Given the context of our findings, it may be critical for the NHIS pro-poor policy to pay special attention to rural residents and those with low or no formal education. While premium exemption for such populations may be tenable, conducting outreach programs that inform rural residents about the benefits of NHIS enrollment is especially important and practical for closing the enrollment gaps. For these outreach programs, it may be useful to design information in local languages and other relatable formats that explain the process of NHIS enrollment and the associated benefits.
